# Supplementary material for: Patterns of Intron Gain and Loss in Fungi
Source: PLoS Biol. 2004 Nov 30;2(12):e422. doi: 10.1371/journal.pbio.0020422 (PMC532390; doi:10.1371/journal.pbio.0020422)
Supplement: Table S1 — Also available at http://genes.mit.edu/NielsenEtAl/. (4.3 MB ZIP). [file pbio.0020422.st001.zip › NielsenEtAl/html/508.html]

AN7741.1.NCU09673.1.MG05484.1.FG02105.1


```
 CLUSTAL W (1.82) Multiple Sequence Alignments - Introns Inserted


Sequence 1: MG05484.1	437 aa
Sequence 2: FG02105.1	436 aa
Sequence 3: NCU09673.1	436 aa
Sequence 4: AN7741.1	454 aa
Alignment Length: 454 aa
Number Identitical Residues: 351 aa
Alignment Score (without introns) 14283


MG05484.1 	MLSGVLLFNLKGENLIFRSFRNDCRPRLADVFRIQVISARETRSPILTLGSTTFSHVKHE
NCU09673.1	MLSGILIFNQKGENLIFRAFRNDCRPRLADVFRIQVISNAQVRSPILTLGSTTFSHVKHE
FG02105.1 	MLSGVLVFNQKGENLIFRAFRNDCRPRLADVFRIQVISNAQVRSPILTLGSTTFSHVKHE
AN7741.1  	MLSGILIFNQKGENLIFRAFRNDCRPRLADIFRIQVISNPQVRSPILTLGSTTFSHVKHE
          	****:*:** ********:***********:*******  :.******************

MG05484.1 	NIYLVAITKSNANAALVFEFLYRLIQLGRGYFGKFDEEAVKNNFVLVYELLDE1IVDFGY
NCU09673.1	NIYLVAITRSNANAALVFEFLYRLIQLGRGYFGKFDEEAVKNNFVLVYELLDE1IIDFGY
FG02105.1 	NIYLVAITKSNANAALVFEFLYRLIQLGKGYFGKFDEEAVKNNFVLVYELLDE1IIDFGY
AN7741.1  	NIYLVAVTKSNANAALVFEFLYRLVLLGKSYFGKFDEEAVKNNFVLIYELLDE1ILDFGY
          	******:*:***************: **:.****************:****** *:****

MG05484.1 	PQNTETDTLKMYITTEGVKSEHR--PEDSAKITMQATGALSWRKADVKYRKNEAFVDVIE
NCU09673.1	PQNTETDTLKMYITTEGVKSERA--VEDSAKITMQATGALSWRKADVKYRKNEAFVDVIE
FG02105.1 	PQNTETDTLKMYITTEGVKSESR--KEDTSKITMQATGALSWRKADVKYRKNEAFVDVIE
AN7741.1  	PQNTETDTLKMYITTEGVKSAITNNPSDSARITQQATGALSWRRADVKYRKNEAFVDVIE
          	********************   .. .*:::** *********:****************

MG05484.1 	DVNLLMSAAGAVLRADVTGQIVMRAYLSGTPECKFGLNDRLLLDGDGMMSLPS-----GN
NCU09673.1	DVNLLMSATGSVLRADVNGQIIMRAYLSGTPECKFGLNDRLLLDQDGLMSLPS-----GN
FG02105.1 	DVNLLMSATGAVLRADVTGQIIMRAYLSGTPECKFGLNDRLLLDNDGLLSLPS-----GN
AN7741.1  	DVNLLMSATGTVLRADVNGQIVMRAYLSGTPECKFGLNDRLLLDNDAAGPGSSNPGAGGR
          	********:*:******.***:********************** *.  . .*...:.*.

MG05484.1 	RMG---SKATKAAAGSVTLEDCQFHQCVKLGKFDSDRIISFVPPDGEFELMRYRATENVN
NCU09673.1	RMG---SKATKAAAGSVTLEDCQFHQCVKLGKFDSDRIISFIPPDGEFELMRYRATENVN
FG02105.1 	KMG---TKATKAAAGSVTLEDCQFHQCVKLGKFDADRIISFVPPDGEFELMRYRATENVN
AN7741.1  	GVGGHSSSKTRAAAGSVTLEDCQFHQCVKLGRFDSDRIISFVPPDGEFELMRYRATENVN
          	 :*. ::. *:********************:**:******:******************

MG05484.1 	LPFKVHAIVNEIGKTKVEYSIGVKANFGPKLFATNVIVRIPTPLNTARITERCTQGKAKY
NCU09673.1	LPFKVHAIVNEVGKTKVEYSIGVRANFGSKLFATNVVVKIPTPLNTARITERCTQGKAKY
FG02105.1 	LPFKVHAIVNEVGRSKVEYSIGVKANFGSKLFATNVIVKIPTPLNTAKITERCTQGKAKY
AN7741.1  	LPFKVHPIVREIGTTKVEYSVAIKANYSSKLFATNVVIRIPTPLNTAKTTERTSQGRAKY
          	******.**.*:* :*****:.::**:..*******:::********: *** :**:***

MG05484.1 	EPSENHIIWKIGRFAGQSEYVLSAEAELTSMTSQKAWSRPPLSMNFSLLMFTSSGLLVRY
NCU09673.1	EPSENVIVWKIGRFAGQSEFVLSAEAELTSMTNQKAWSRPPLSMNFSLLMFTSSGLLVRY
FG02105.1 	EPSENVIIWKIGRFTGQSEYVLSAEAILTSMTEQRAWSRPPLSMNFSLLMFTSSGLLVRY
AN7741.1  	EPEHNNIVWKIARFSGGSEYVLTAEATLSAMTNQKAWSRPPLSLNFSLLMFTSSGLLVRY
          	**..* *:***.**:* **:**:*** *::**.*:********:****************

MG05484.1 	LKVFEKSNYSSVKWVRYLSRAGSYEIRF-------
NCU09673.1	LKVFEKSNYSSVKWVRYMTRAGSYEIR--------
FG02105.1 	LKVFEKSNYSSVKWVRYMTRAGSYEIR--------
AN7741.1  	LKVFEKSNYSSVKWVRYMTRAGSYEIRYVYFECSW
          	*****************::********    ..:
```
